# Supplementary material for: Mechanofusion-derived cathode composite microstructures with scalable mixed conducting matrix coatings for solid state batteries
Source: Nat Commun. 2026 Apr 3;17:3215. doi: 10.1038/s41467-026-71305-2 (PMC13056986; doi:10.1038/s41467-026-71305-2)
Supplement: Supplementary file 1 — Supplementary Information [file 41467_2026_71305_MOESM1_ESM.pdf]

Supplementary Information for

Mechanofusion-derived cathode composite microstructures with scalable mixed conducting matrix coatings for solid state batteries

Maximilian Kissel[1,†], *Finn Frankenberg*[2,†], *Thomas Demuth*[3], *Anton Lai*[1], *Niklas Laser*[2], *Daniel Wagner*[1], *Ahmed Eisa*[2], *Peter Michalowski*[2], *Kerstin Volz*[3], *Arno Kwade*[2,*], *Jürgen Janek*[1,*]

[1]Justus-Liebig-Universität Gießen, Institute of Physical Chemistry & Center for Materials Research, Heinrich-Buff-Ring 17, Gießen, 35392, Hesse, Germany

[2]Technische Universität Braunschweig, Institute for Particle Technology, Volkmaroder Straße 5,
Braunschweig, 38104, Lower Saxony, Germany

[3]Materials Science Center (WZMW) and Department of Physics, Philipps-University Marburg, Marburg 35032, Hesse, Germany

*Corresponding authors:

E-mail: juergen.janek@pc.jlug.de; arno.kwade@tu-braunschweig.de

† These authors contributed equally to this work

Table S1. Overview about the exact weighing and the calculated mixed densities.

Type of Investigation	Nomenclature in manuscript	Composition (Mass in g)				Composition (Volume in cm^3)				Mixed density / g cm^{-3}
		NCM	LIC		∑	NCM	LIC		∑	ρ_{mix}
Coating Thickness	99:1:0	22.92	0.23		23.15	4.82	0.09		4.91	4.71
Variation (**without** CB)	98:2:0	22.49	0.46		22.95	4.73	0.18		4.91	4.67
Variation of coating	95:5:0	21.28	1.12		22.40	4.48	0.43		4.91	4.56
content 1-20 wt.%	90:10:0	19.35	2.15		21.50	4.07	0.83		4.90	4.38
	80:20:0	16	4		20.00	3.37	1.54		4.91	4.07
		NCM	LIC	CB	∑	NCM	LIC	CB	∑	ρ_{mix}
Coating Thickness	99:1:0.2	22.83	0.23	0.04	23.1	4.81	0.09	0.02	4.91	4.70
Variation (**with** CB)	98:2:0.3	22.32	0.46	0.07	22.85	4.7	0.18	0.04	4.91	4.65
Variation of coating	95:5:0.8	20.93	1.1	0.17	22.2	4.41	0.43	0.09	4.92	4.51
content 1-20 wt.%	90:10:1.5	18.76	2.08	0.32	21.17	3.95	0.8	0.16	4.92	4.3
	80:20:3	15.05	3.76	0.58	19.4	3.17	1.45	0.3	4.92	3.94
		NCM	LIC	CB	∑	NCM	LIC	CB	∑	ρ_{mix}
Process Parameter	95:5:0.8	20.93	1.1	0.17	22.2	4.41	0.43	0.09	4.92	4.51
Variation	80:20:3	15.05	3.76	0.58	19.4	3.17	1.45	0.3	4.92	3.94
		NCM	LIC	CB	∑	NCM	LIC	CB	∑	ρ_{mix}
Matrix Optimization	80:20:0	16	4	0	20.00	3.37	1.54	0	4.91	4.07
CB content variation:	80:20:0.2	15.93	3.98	0.04	19.95	3.35	1.54	0.02	4.91	4.06
0-3 w.t%	80:20:0.5	15.84	3.96	0.1	19.9	3.33	1.53	0.05	4.91	4.05
	80:20:1	15.68	3.92	0.2	19.8	3.3	1.51	0.1	4.92	4.03
	80:20:2	15.35	3.84	0.39	19.58	3.23	1.48	0.2	4.91	3.99
	80:20:3	15.05	3.76	0.58	19.4	3.17	1.45	0.3	4.92	3.94

Supplementary Note 1 - Particle Size Distributions via particleOS.ai

The SEM Image Analysis Tool, part of the particleOS.ai platform developed at the Institute for Particle Technology at Technical University Braunschweig, enables quantitative characterization of scanning electron microscope images through AI-driven segmentation and morphological analysis. Meta's Segment Anything Model (SAM) provides precise particle boundary delineation through two operational modes: automatic segmentation for batch processing of multiple particles, and manual selection mode for targeted analysis of specific regions of interest. The workflow consists of image upload, scale calibration, and mode selection, followed by automated segmentation and Morphological analysis.

The system generates number-based (q_0) particle size distributions by calculating equivalent circle diameter ($\sqrt{(4\times\text{Area}/\pi)}$). Users can analyze multiple SEM images of the same sample and automatically generate averaged distributions for improved statistical robustness.

Morphological characterization includes calculation of key shape metrics, particularly circularity ($4\pi \times$Area/Perimeter^2) and aspect ratio (maximum Feret diameter/minimum Feret diameter). Circularity quantifies how closely a particle resembles a perfect circle (value of 1.0), while aspect ratio identifies elongated particles. Additional parameters including convexity, solidity, and equivalent diameter are also calculated. These metrics enable classification into distinct morphological categories and provide quantitative data on shape heterogeneity.

This implementation improves upon previous approaches [1] by leveraging SAM's zero-shot capabilities with customized filtering algorithms. As demonstrated in the manual mode analysis above, this eliminates the need for specialized training datasets while improving the processing of complex, overlapping particle arrangements that are challenging for conventional segmentation methods.

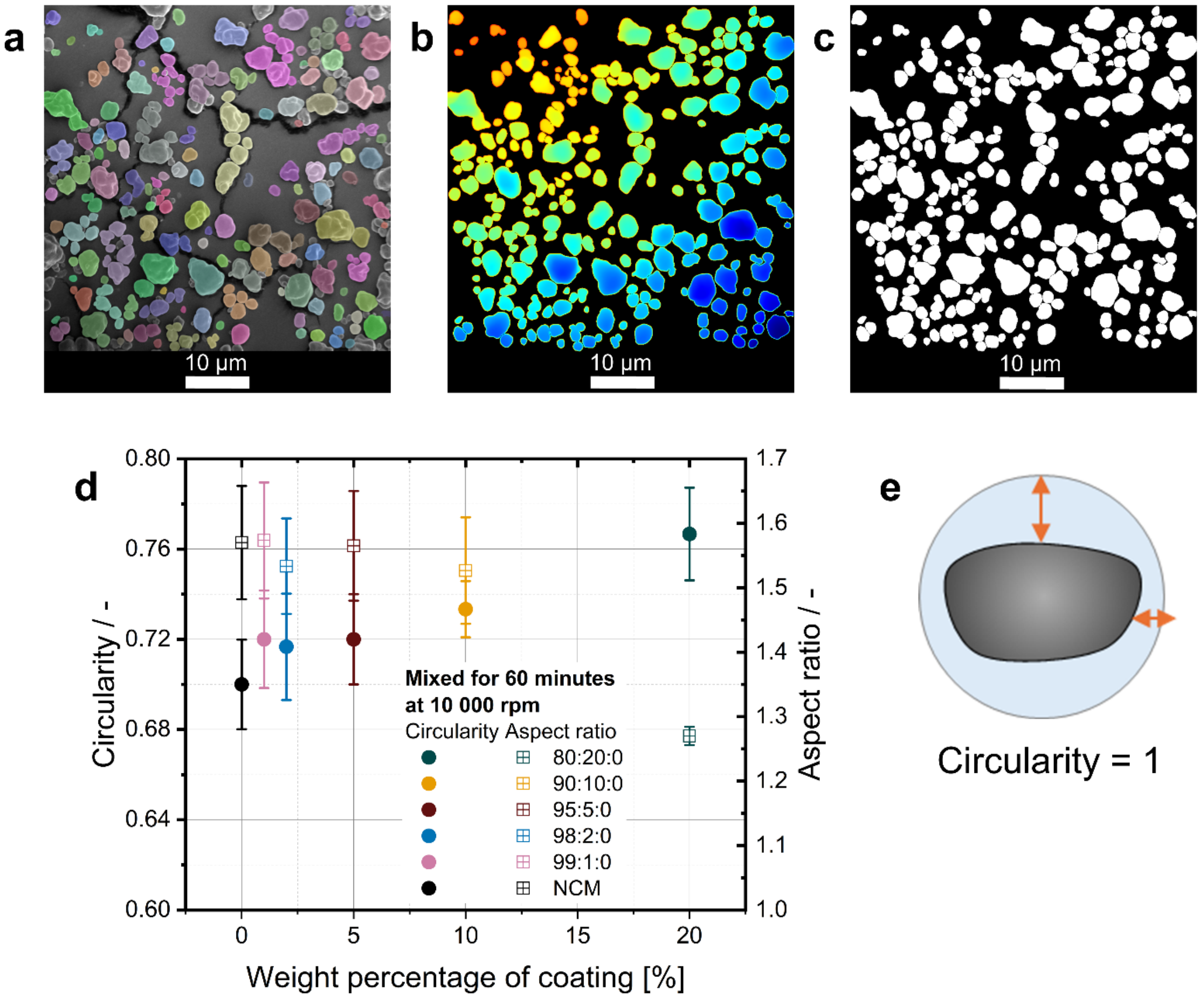

Figure S1. *Exemplary result from the in-house particleOS.ai tool, which was used for analyzing the coating based on SEM images.* ***a*** *Example of a segmented SEM image of the 80:20:0 composition, accompanied by the corresponding depth map* ***b*** *and binary image* ***c****. In addition to the PSD, particle shape descriptors such as circularity and aspect ratio were determined with the particleOS.ai tool, as illustrated in* ***d*** *for different coating compositions. Error bars represent the standard deviation of n = 3 segmented SEM images.* ***e*** *Simplified sketch illustrating that a high circularity value means that the NCM particle is covered by a differently thick coating layer given the non-spherical shape of the NCM particle.*

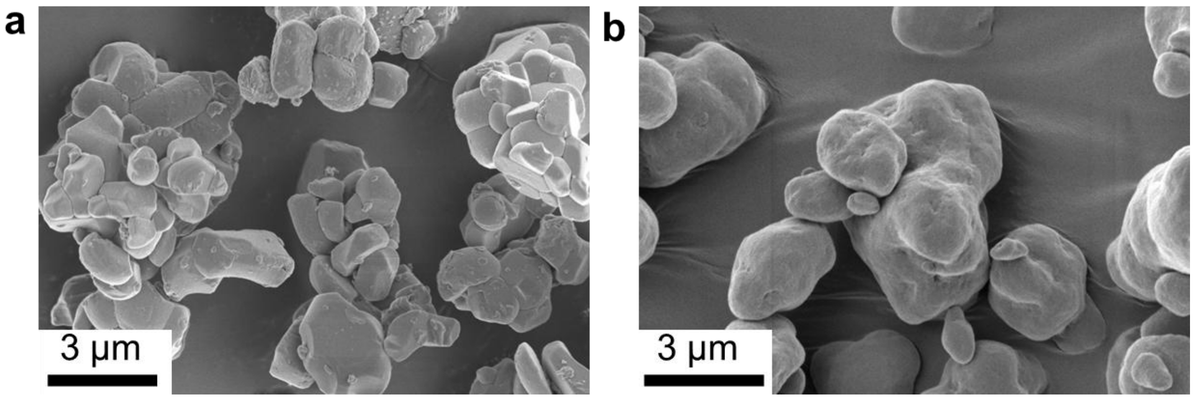

Figure S2. *SEM images of the* ***a*** *pristine NCM and* ***b*** *LIC-coated NCM (80:20:0).*

Supplementary Note 2 – Calculation of the theoretical coating thickness

The theoretical coating thickness was calculated using the raw material densities, the mixed density of the coated material, and geometric considerations. As a first step, the average mass and volume of one NCM particle was approximated based on the median particle diameter of 2.05 µm obtained from the q_0 PSD.

$$V_{\mathrm{NCM}}=\frac{4}{3}\pi \cdot R_{\mathrm{NCM}}^3$$

$$m_{NCM}=V_{\mathrm{NCM}} \cdot \rho_{\mathrm{NCM}}$$

Based on the material compositions and weight fractions, and assuming a uniform coating on each particle, the coating volume per individual NCM particle was calculated. For samples without CB, a coating density of 2.59 g cm^{-3} was used corresponding to the density of pure LIC. For samples containing CB, a reduced coating density of 2.48 g cm^{-3} was applied, calculated from the fixed volume ratio between CB and LIC (20:80 vol.%).

$$V_{\mathrm{coating}}=\frac{m_{\mathrm{coating}}}{\rho_{\mathrm{coating}}}=\frac{wt.\%_{\mathrm{coating}} \cdot m_{\mathrm{NCM}}}{\rho_{\mathrm{coating}}}$$

Based on geometric considerations and using the radius of the NCM particles, the coating thickness can be approximated mathematically. Therefore, the coating volume is derived geometrically as follows:

$$V_{\mathrm{coating}}=\frac{4}{3} \cdot \pi \cdot \left(R_{\mathrm{total}}^3\text{-}R_{\mathrm{NCM}}^3\right)$$

$$V_{\mathrm{coating}}=\frac{4}{3} \cdot \pi \cdot ((s+R_{\mathrm{NCM}})^3\text{-}R_{\mathrm{NCM}}^3)$$

By rearranging this equation for the coating thickness s and substituting the coating volume derived from the volume ratios, the theoretical coating thickness can be calculated.

$$s=\sqrt[3]{\frac{V_{coating} \cdot 3}{\pi \cdot 4}+R_{NCM}^3}\text{-}R_{NCM}$$

The resulting thickness values are depicted in Figure S3:

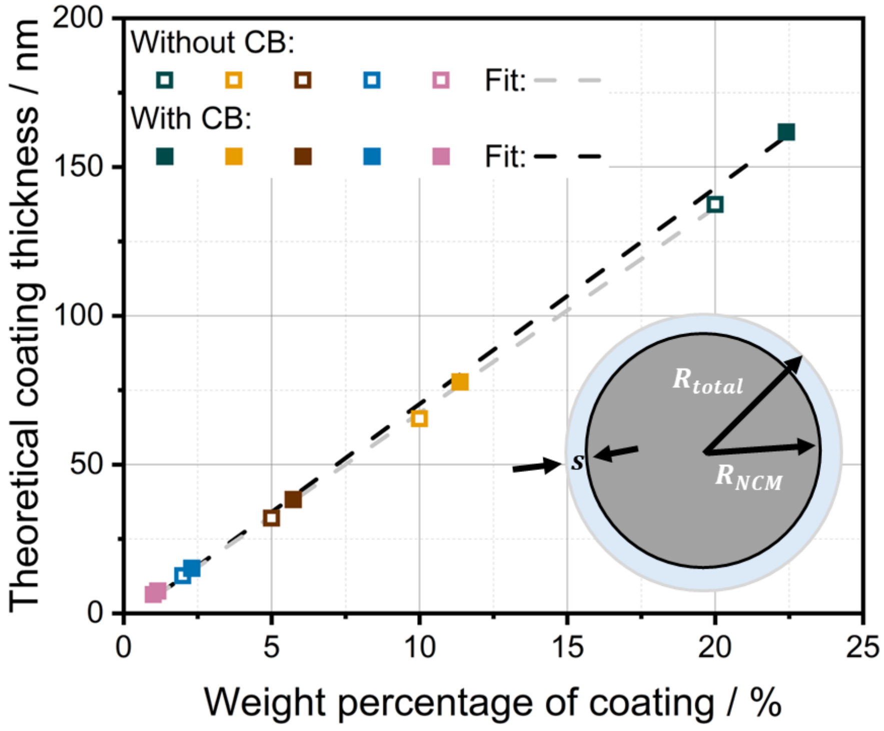

Figure S3. *Theoretical coating thickness depending on coating content. The coating thickness was estimated based on the* d_{50} *value of 2.05 µm* (q_0 *distribution of NCM) and the corresponding weight ratio of the coating material. The calculation assumes spherical NCM particles, uniform coating of each particle and the absence of porosity within the coating layer.*

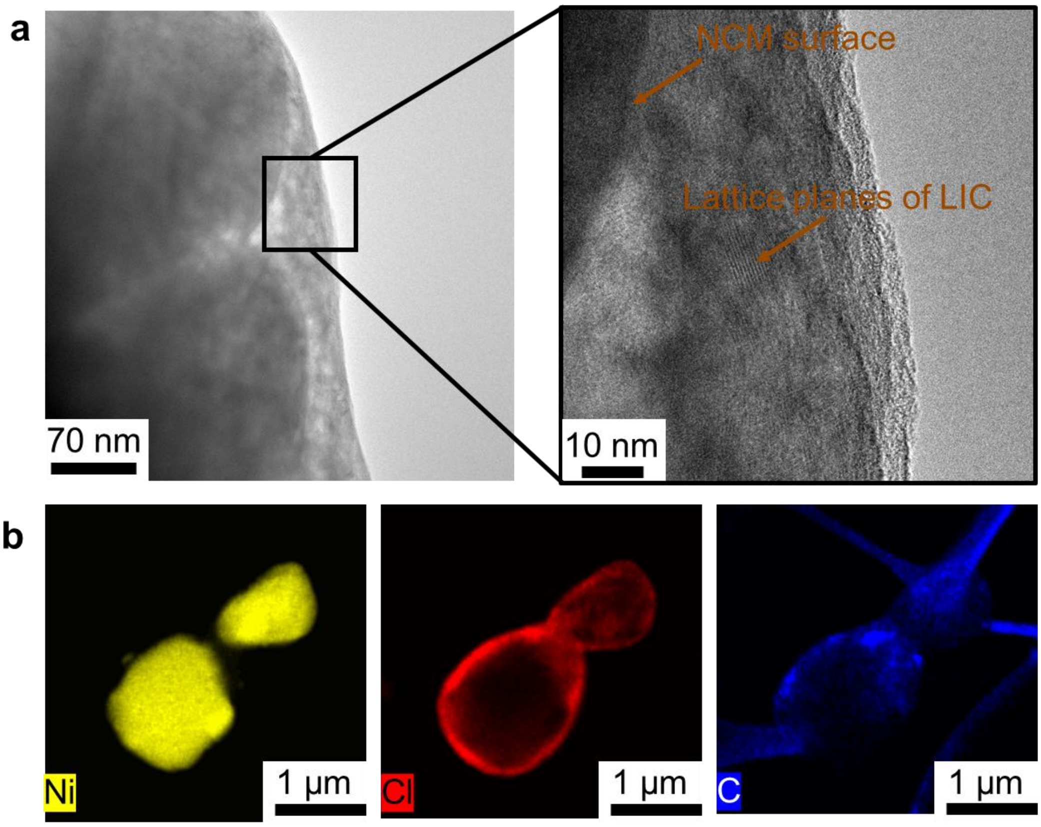

Figure S4. a *HR-TEM images and* ***b*** *STEM-EDX maps of building blocks with 80:20:1 composition.*

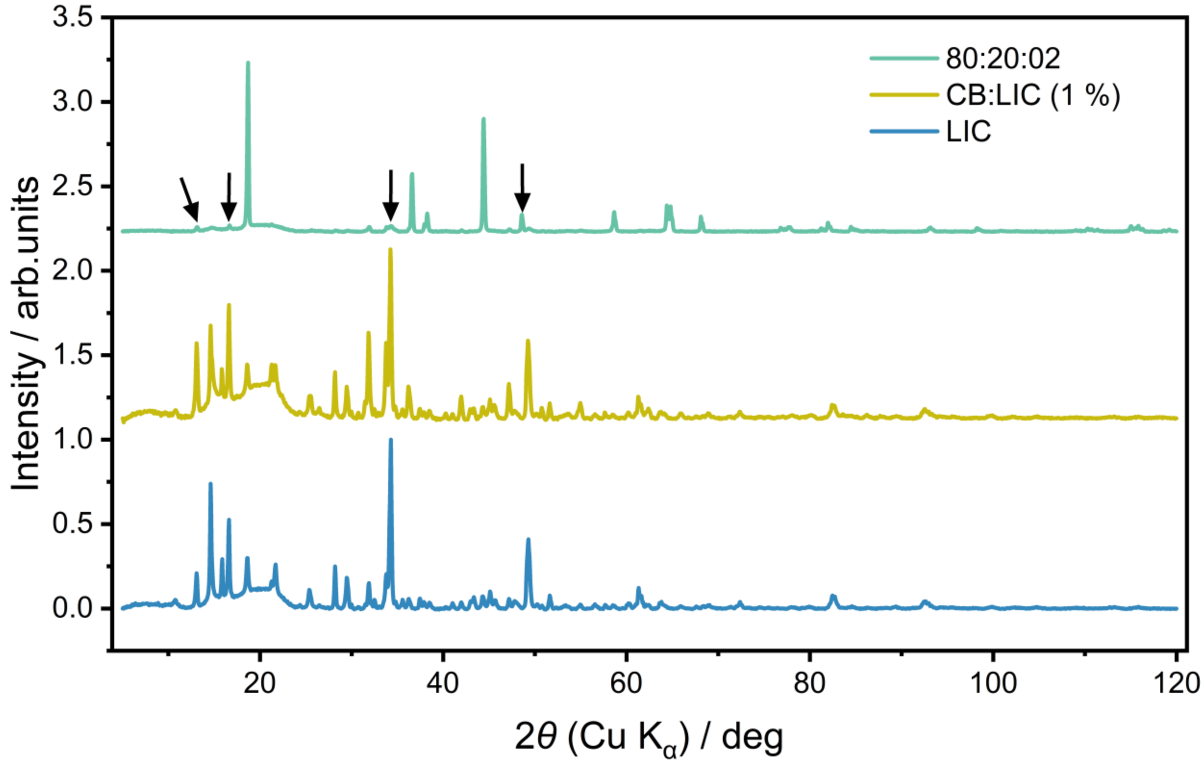

Figure S5. *X-ray diffractograms obtained in reflection geometry of pristine LIC, CB-LIC premix and NCM-LIC-CB composite revealing the presence of LIC in the premix as well as in the composite.*

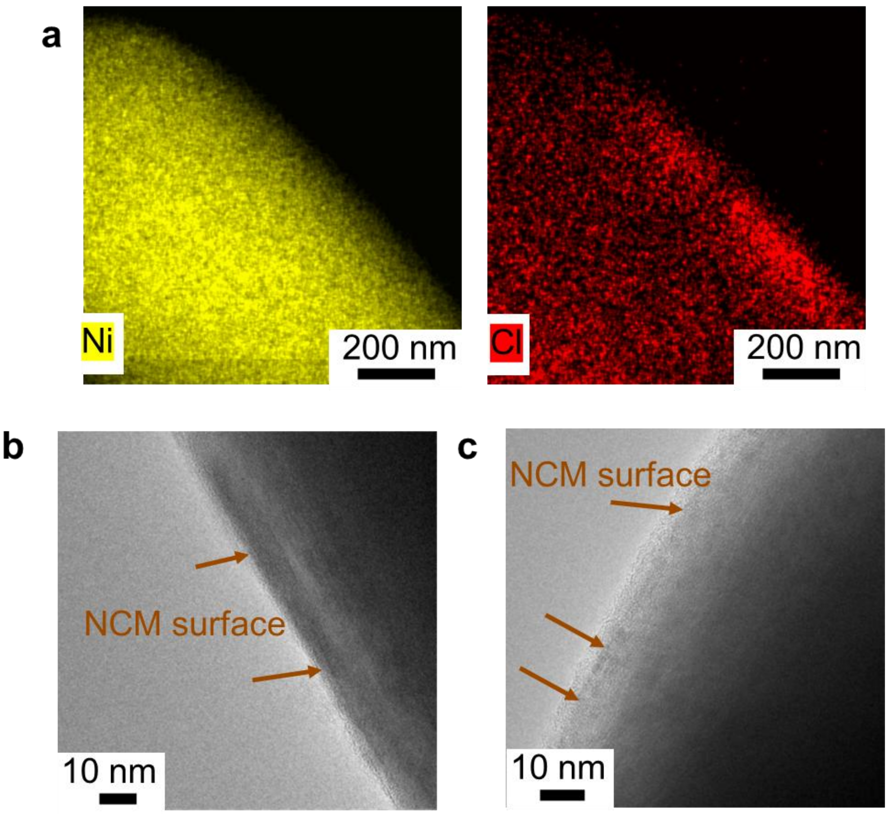

Figure S6. a *STEM-EDX maps and* ***b,c*** *HR-TEM images of coated particles with composition 99:1:0.*

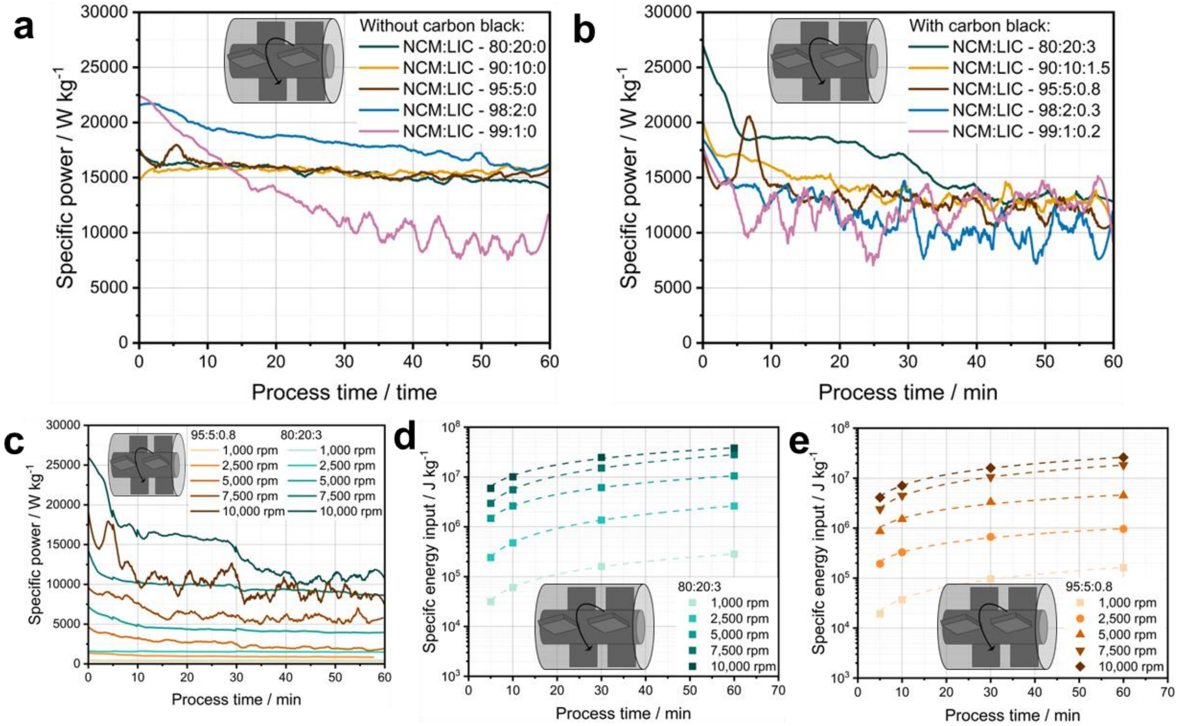

Figure S 7. *Specific power for different compositions* ***a*** *with CB and* ***b*** *without CB.* ***c*** *Specific power recorded for compositions 95:5:0.8 and 80:20:3 depending on the rotational speed. Specific energy input calculated from the recorded power data for the mixing of the compositions* ***d*** *95:5:0.8 and* ***e*** *80:20:3.*

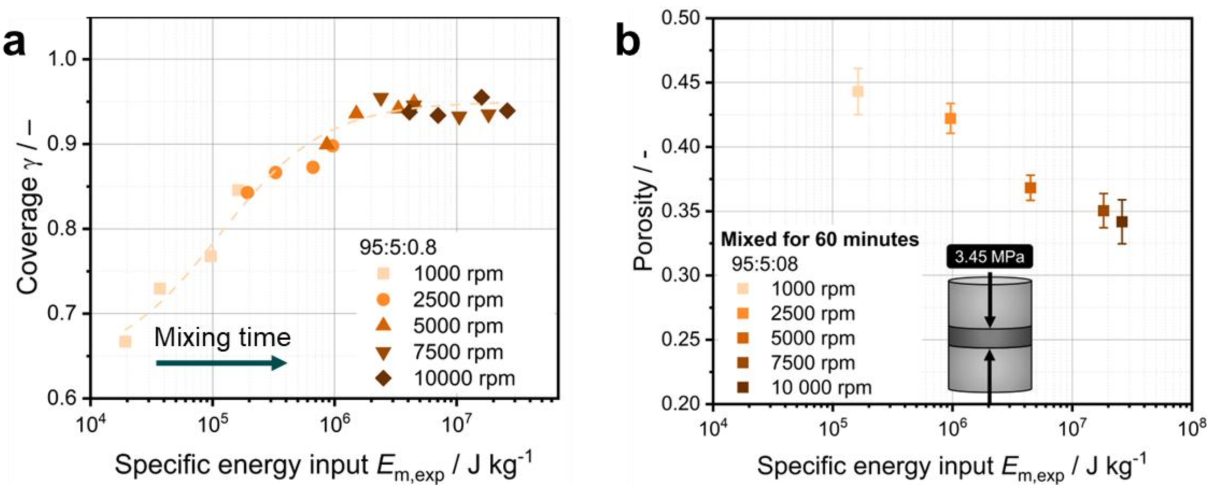

Figure S8. *Evolution of* ***a*** *coverage and* ***b*** *porosity as function of specific energy input during high-intensity mixing. Error bars represent the standard deviation of n = 3 measurements.*

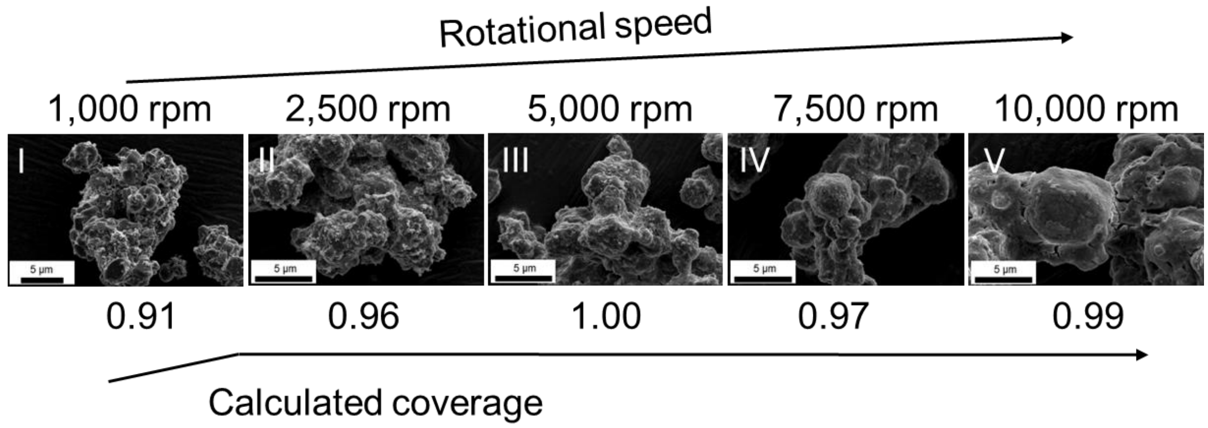

Figure S9. *Calculated coverage values and corresponding SEM images for building blocks with composition 80:20:3 mixed at different rotational speeds.*

Supplementary Note 3 – Calibration and calculation of the coarse grain density

The particle density used in the simulation was calculated to 2.417 g cm^{-3}, based on the measured bulk density of the 80:20:3 powder mixture (1.450 g cm^{-3}), as well as the true material density of the 80:20:3 composition, which is 3.944 g cm^{-3}. Here an internal coarse-grain porosity of 0.39 is assumed.

The simulation was further validated by plotting the coverage as a function of the experimental and simulated specific energy input, fitting both datasets, and calculating the relative error between the resulting curves (cf. Supplementary Figure S11). The maximum deviation was found to be maximum 2 %, demonstrating that the DEM simulation accurately captures the coating process on a macroscopic scale.

It should be noted that by employing the normalized parameters $\overline{SI}_{\mathrm{CG}}$ and $\overline{SN}_{\mathrm{p}}$, the coarse-graining of particles in the DEM simulation is effectively accounted for, as opposed to using the absolute values of the stressing energy $\overline{SE}_{\mathrm{CG}}$ and stress number SN_{CG}. In principle, the $\overline{SI}$ should remain constant when changing the particle size, as both the collision energy and the particle mass under impact stress scale with the cube of the particle diameter ($\overline{SI}_{\mathrm{CG}} = \frac{\overline{SE}_{\mathrm{CG}}}{m_{\mathrm{particle,CG}}} = \frac{\overline{SE}_0 \cdot f^3}{m_{\mathrm{particle,0}} \cdot f^3}$). However, due to the narrow gap between the rotor and chamber wall, the $\overline{SE}_{\mathrm{CG}}$ may be slightly overestimated for the coarse-grained particles. If the scaling of $\overline{SE}_0$

exceeds f^3, this would lead to a minor overestimation of the stress intensity in the coarse-grained system. This is why it is denoted as $\overline{SI}_{\mathrm{CG}}$ throughout this study. The mean stress number per particle should also be scale-invariant, as the stress frequency decreases approximately with the cube of the particle diameter [2], while the coarse-grained particle mass increases with the cube of the particle diameter.

Table S2. *Overview about the simulation parameters applied in this study. See Supplementary Note 3 for details on the particle density.*

Calibration parameters	Particle – Particle	Particle – Wall	
Static friction / -	0.46	0.45	
Dynamic friction / -	0.31	0.25	
Yield ratio / -	0.00756	0.0256	
Restitution coefficient / -	0.3	0.3	
**Properties**	**Particle**	**Wall (Rotor)**	**Wall (Chamber)**
Density / g·cm^{-3}	2.417*	4.510	7.850
Young's modulus / GPa	0.228	120	200
Poisson ratio / -	0.3	0.3	0.3

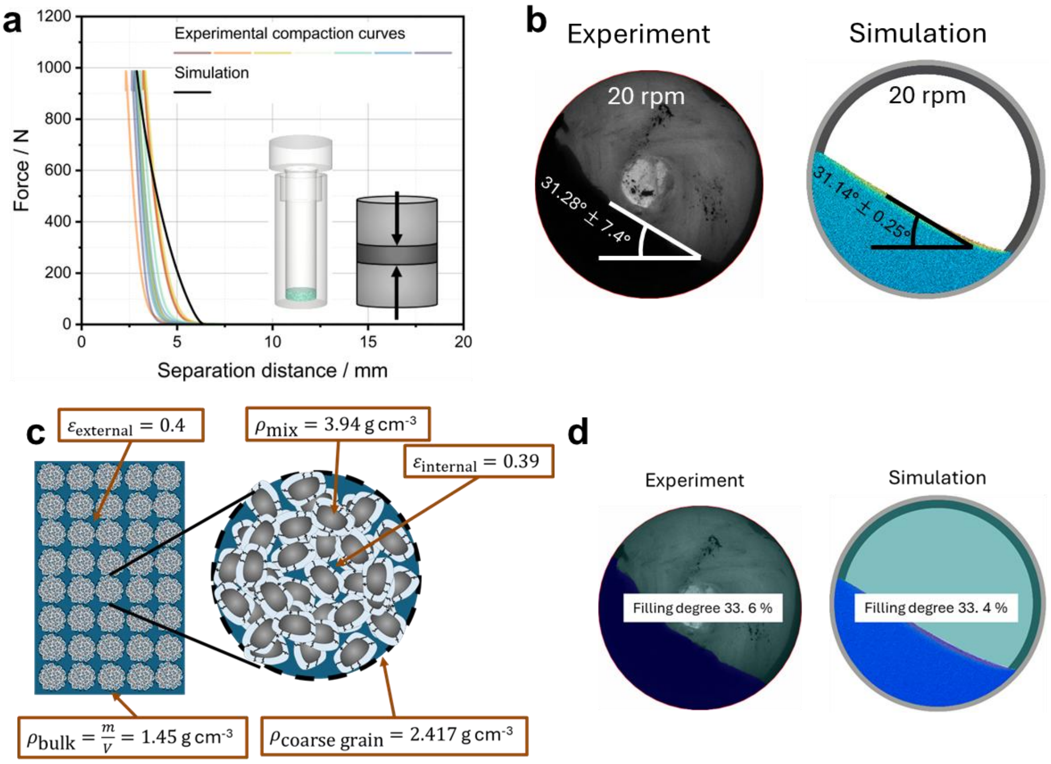

Figure S10. *Calibration procedure of the DEM simulation with* ***a*** *compaction test simulation and experiment,* ***b*** *dynamic angle of repose simulation and experiment,* ***c*** *illustration of how the coarse-grain density was calculated and* ***d*** *comparison of the filling degree between simulation and experiment, thereby confirming the coarse-grain density.*

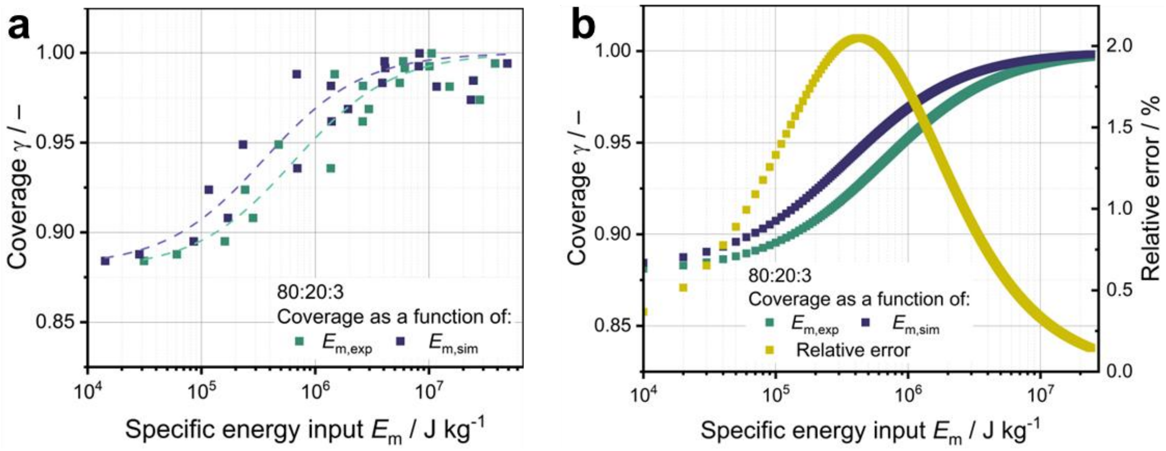

***Figure S11. a** Coverage as a function of the experimental and simulated specific energy input and their fit functions and **b** fit functions from the coverage and the corresponding relative error.*

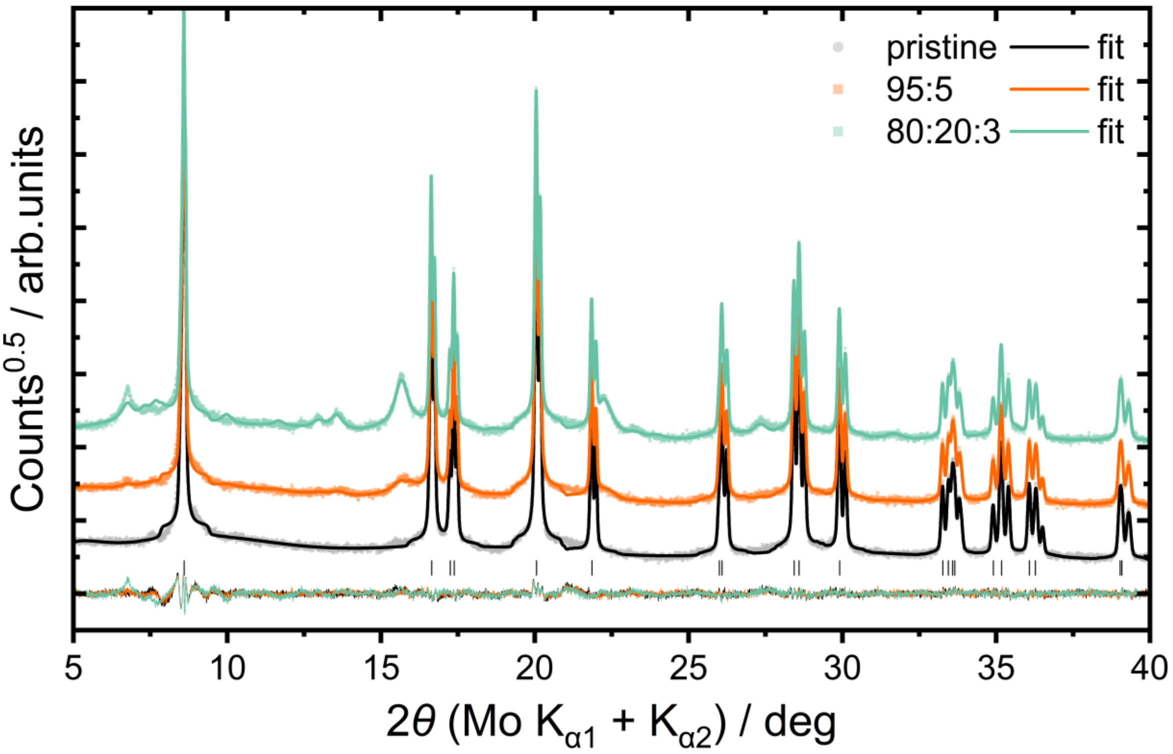

***Figure S12.** X-ray diffractograms obtained in transmission geometry of pristine NCM and two LIC-coated NCM samples with a thin (95:5) and a thick (80:20:3) coating.*

Table S3. *Lattice parameters, lattice strain, and mass ratios out of Rietveld refinement of three exemplary samples.*

Sample	a / Å	c / Å	Lattice strain / %	NCM / wt.%	LIC / wt.%
Pristine	2.895739	14.304153	0.037	100	-
95:5:0	2.895918	14.304236	0.05	92	8
80:20:3	2.895848	14.303675	0.045	79	21

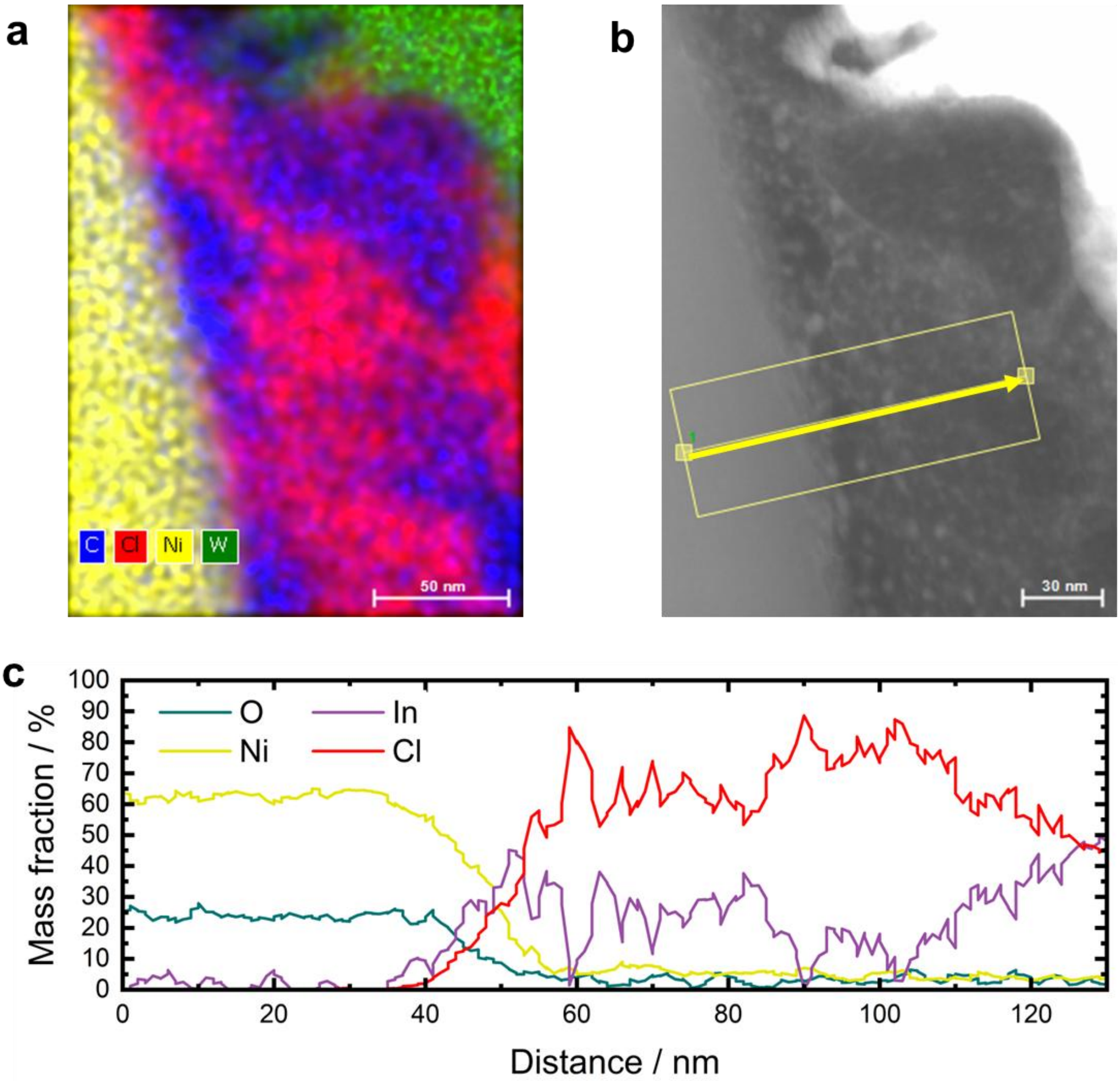

Figure S 13. *STEM-EDX line scan.* ***a*** *Elemental map,* ***b*** *line scan direction and* ***c*** *measured mass fraction versus distance curve. Due to the NCM surface being slightly slanted, both materials overlap a bit in this region. However, no sign of element diffusion beyond this overlapping area of approximately 10-15 nm width was detected.*

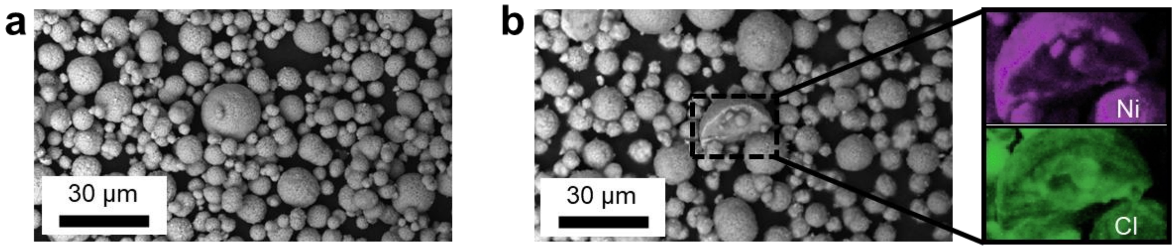

Figure S* 14.** *SEM images of* ***a *polycrystalline NCM raw material and* ***b*** *polycrystals after processing at 10,000 rpm for 60 min with 20 wt.% LIC.*

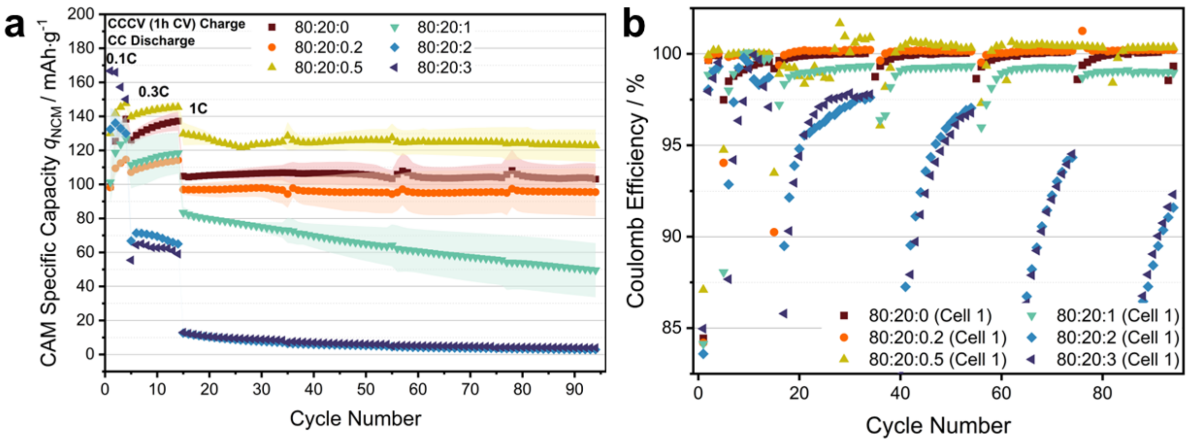

Figure S 15. *Cycling performance depending on the cathode composition.* ***a*** *CAM-specific capacity (using the total nominal CAM mass in the cathode) versus cycle number when cycled in CCCV mode at 25°C under 80 MPa stack pressure (1C ≈ 200 mA/g ≈ 3 mA/cm²). Error bars represent the standard deviation of n = 2 cells.* ***b*** *Corresponding Coulomb efficiencies (CEs) versus cycle number. See Supplementary Note 4 for additional comments.*

Supplementary Note 4 – Periodicity in Coulomb efficiency

In Figure S15a fluctuations in the capacities are observed every 20 cycles during 1C CCCV cycling. Furthermore, a periodicity in Coulomb efficiencies (CEs) over 20 cycles at 1C is observed in Figure S15b, most obvious for compositions 80:20:2 and 80:20:3. Thereby a characteristic drop in CE is observed followed by a gradual recovery over the subsequent cycles.

Both phenomena are directly related to the determination of the CAM utilization every 20 cycles. This step, referred to as the check-up cycle in the following, involves slower charging and discharging as well as extended equilibration periods (see Methods section). As a result, it

deviates significantly from the monotonic high C-rate CCCV cycling, leading to periodic fluctuations in both capacities and CEs. Importantly, this step alters the lithium inventory within the CAM, increasing its lithium content at the beginning of each 20-cycle sequence. In the first cycle after the check-up, this ‘excess’ lithium inventory is exploited, leading to higher extracted charge. During subsequent discharging, the same amount of lithium must be reinserted to maintain a high CE. However, for compositions such as 80:20:2, kinetic limitations dominate, resulting in incomplete re-lithiation and thus a temporary CE reduction. Over the following cycles, the CAM remains less lithiated than at the beginning, which reduces the kinetic asymmetry between discharge and charge and leads to a gradual recovery of CE.

For the 80:20:0.5 composition, CEs above 100% are observed over several cycles, which at first glance appears unphysical. However, the CE in the first cycle of each 20-cycle block is clearly below 100%. In the subsequent cycles, the remaining Li sites are progressively refilled, resulting in apparent CEs > 100%. When the total charge and discharge capacities of all 20 cycles are considered together, the cumulative CE remains below 100% (99.32 % for the 80:20:0.5 cell), which is physically reasonable.

Overall, the extended rest period during the check-up cycle allows the system to equilibrate, resulting in an asymmetric kinetic response between discharge and charge. This imbalance is reinforced immediately after the check-up cycle.

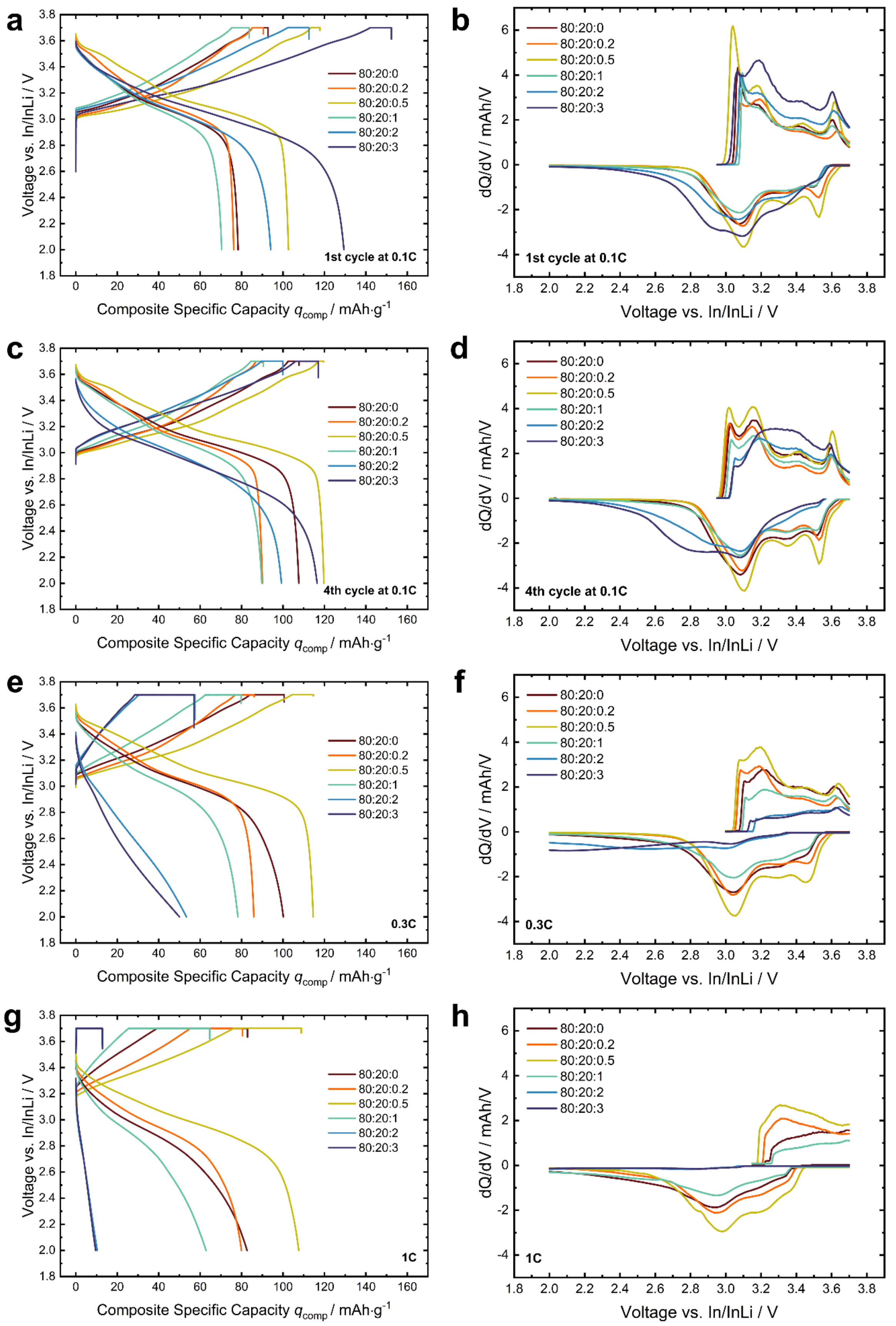

Figure S 16. *Full voltage curves and differential charge plot of all investigated compositions for the* ***a,b*** *initial cycle at 0.1C,* ***c,d*** *the last cycle at 0.1C,* ***e,f*** *a representative cycle at 0.3C and* ***g,h*** *a representative cycle at 1C (1C ≈ 200 mA/g ≈ 3 mA/cm²).*

Table S4. *Electrochemical data for all compositions: Initial charge capacities $Q_{0,\text{charge}}$ of the CC and CV step, initial discharge capacities $Q_{0,\text{discharge}}$ (all referred to the total composite mass), initial Coulomb efficiency and capacity retention over 80 cycles at 1C in CCCV mode.*

Composition	$Q_{0,\text{charge}}$ (CC) mAh·g^{-1}	$Q_{0,\text{charge}}$ (CV) mAh·g^{-1}	$Q_{0,\text{discharge}}$ (CC) mAh·g^{-1}	ICE %	Capacity retention %
80:20:0	85.3	7.5	78.3	84.4	91.7
80:20:0.2	85.2	5.3	76.2	84.2	108.5
80:20:0.5	113.0	4.8	102.6	87.1	96.7
80:20:1	75.4	8.3	70.4	84.1	81.8
80:20:2	102.1	10.5	94.1	83.6	21.8
80:20:3	142.0	10.3	129.4	85.0	30.1

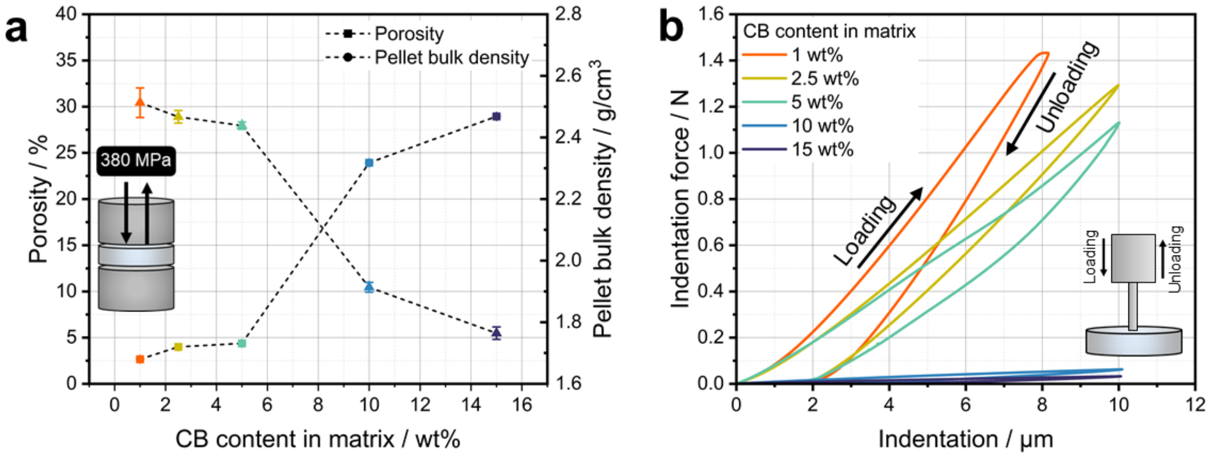

Figure S17. *Investigation of the mechanical properties of the CB-LIC premix.* ***a*** *Porosity and bulk density of pellets prepared from the CB-LIC premix compacted at 380 MPa. Error bars represent the standard deviation of n = 24 indentations.* ***b*** *Nanoindentation measurements performed on the pellets shown in a. Each force–indentation curve represents the average of 25 individual indents.*

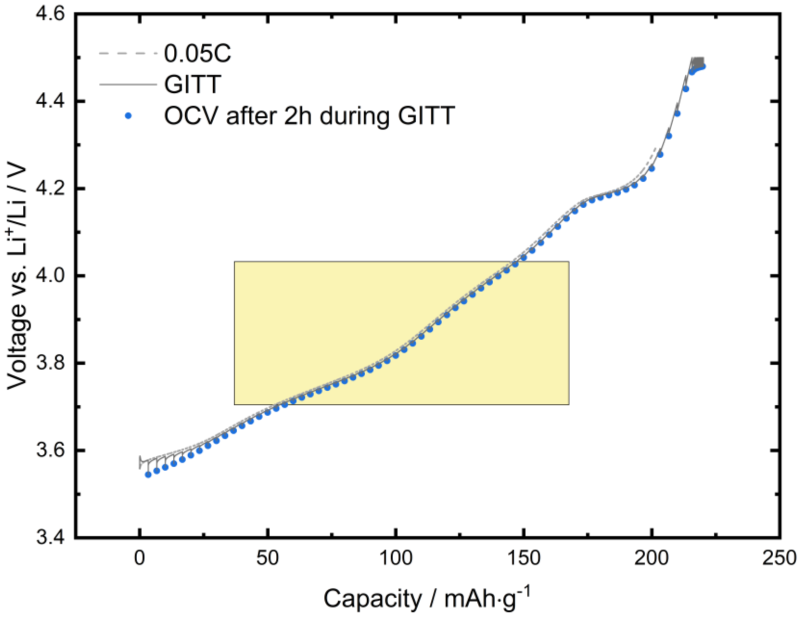

Figure S18. *Comparison between the voltage versus capacity profile obtained from galvanostatic charging at 0.05C and from galvanostatic intermittent titration technique (GITT) for a LIB reference cell. The relaxed OCV after 2h during GITT are displayed as blue data points. The curves significantly overlap, especially in the yellow voltage region, which was used for the CAM utilization determination. The low C-rate charging profile is thus considered as quasi-OCP and used to quantify the CAM utilization in the SSB cells.*

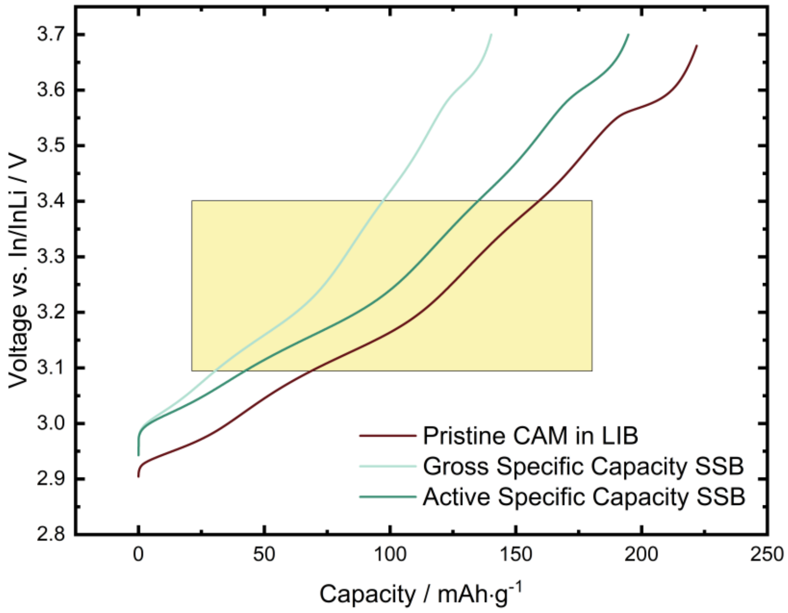

Figure S19. *Comparison of Voltage-Capacity curves for the LIB reference cell and the SSB cell. For the SSB cell, the gross specific capacity, referred to the total mass of CAM in the cathode, and the active specific capacity, referred only to the active fraction of CAM in the cathode are displayed. No significant differences between the voltage curves of the LIB and the SSB, especially in the yellow voltage region, which was used for the CAM utilization determination, are observed. The visible shift is due to kinetic effects since the SSB was cycled at 0.1C while the LIB was cycled a 0.02C.*

Supplementary References

[1] J. Bals, K. Loza, P. Epple, T. Kircher and M. Epple, "Deep learning for automated size and shape analysis of nanoparticles in scanning electron microscopy," RSC Advances, 2023, 13, 2795-2802.

[2] M. Sakai, M. Abe, Y. Shigeto, S. Mizutani, H. Takahashi, A. Viré, J. Percival, J. Xiang, C. Pain, "Verification and validation of a coarse grain model of the DEM in a bubbling fluidized bed," Chemical Engineering Journal, 2014, 244, 33-43.
